# Moderate grazing promotes the root biomass in *Kobresia* meadow on the northern Qinghai–Tibet Plateau

**DOI:** 10.1002/ece3.5494

**Published:** 2019-07-30

**Authors:** Licong Dai, Xiaowei Guo, Xun Ke, Fawei Zhang, Yikang Li, Cuoji Peng, Kai Shu, Qian Li, Li Lin, Guangmin Cao, Yangong Du

**Affiliations:** ^1^ Key Laboratory of Adaptation and Evolution of Plateau Botany, Northwest Institute of Plateau Biology Chinese Academy of Science Xining China; ^2^ University of Chinese Academy of Science Beijing China; ^3^ College of Life Sciences Luoyang Normal University Luoyang China

**Keywords:** functional groups, grazing intensities, plant biomass, productivity–richness relationship, species composition

## Abstract

Grazing is an important modulator of both plant productivity and biodiversity in grassland community, yet how to determine a suitable grazing intensity in alpine grassland is still controversy. Here, we explore the effects of different grazing intensities on plant biomass and species composition, both at community level and functional group level, and examines the productivity–species richness relationship under four grazing patterns: no grazing (CK), light grazing (LG), moderate grazing, (MG) and heavy grazing (HG), attempt to determine a suitable grazing intensity in alpine grassland. The results were as follows. The total aboveground biomass (AGB) reduced with increasing grazing intensity, and the response of plant functional groups was different. AGB of both sedges and legumes increased from MG to HG, while the AGB of forbs reduced sharply and the grass AGB remained steady. There was a significant positive relationship between productivity and species richness both at community level and functional group level. In contrast, the belowground biomass (BGB) showed a unimodal relationship from CK to HG, peaking in MG (8,297.72 ± 621.29 g/m^2^). Interestingly, the grassland community tends to allocate more root biomass to the upper soil layer under increasing grazing intensities. Our results suggesting that moderate levels of disturbance may be the optimal grassland management strategy for alpine meadow in terms of root production.

## 1 INTRODUCTION

Grassland is widely distributed around world, making a great contribution to the balance between forage productivity and livestock (Feng et al., 2010), and playing a vital role in the global carbon cycle (Yang, Fang, Ji, & Han, 2009). Nevertheless, in the past few years, an increasing proportion of grassland has been suffered from processes such as fire, mowing, soil disturbance and grazing (Hobbs & Huenneke, 1992). Among these processes, grazing is known as one of the most important disturbance factors leading to worldwide grassland degradation and the subsequent decline of grassland productivity and ecological function (Sala, Parton, Joyce, & Lauenroth, 1988). If grazing pressure exceeds the carrying capacity of the grassland (i.e., overgrazing), which could severely reduces soil infiltration rates, vegetation cover, grassland productivity and the proportion of forage grasses, resulting in the degradation of topsoil (Hilker, Natsagdorj, Waring, Lyapustin, & Wang, 2014). Thus, understanding the effects of different grazing intensities on both species composition and biomass in an alpine ecosystem could yield new insights into grassland management (Piao, Fang, & He, 2006).

Many studies have documented how overgrazing can alter both pasture production and the composition and structure of grassland (Pulido, Schnabel, Lozano‐Parra, & González, 2016; Török et al., 2018; Tóth et al., 2018). For instance, the regenerative ability of grasslands was significant reduced, and the physical and chemical properties of soil were clearly altered (for example soil bulk density, soil water content and soil nutrients) following overgrazing (Byrnes, Eastburn, Tate, & Roche, 2018; Lin et al., 2015). There is growing evidence that soil bulk density and soil moisture content, respectively, increase and decrease significantly, after long‐term heavy grazing (Hofstede, 1995; Lu et al., 2017). In addition, a previous study indicated that the organic carbon content decreased with increasing grazing intensity (Wang, Zhang, Chen, Tan, & Sun, 2010). Overall, the impacts of grazing on soil condition have been well documented in previous studies, whereas no consensus has been obtained regarding the effect of grazing intensity on species composition and grassland productivity. For instance, several studies have shown that species richness and ANPP were significantly reduced in grazed areas relative to ungrazed areas (Lin et al., 2015; Lu et al., 2017). In contrast, other studies have observed greater species richness and higher ANPP in continuously grazed areas when compared with ungrazed areas (Altesor, Oesterheld, Leoni, Lezama, & Rodríguez, 2005; Altesor et al., 2006), and a positive relationship was also found between ANPP and species richness in grazed areas (López Mársico & Altesor, 2011). Meanwhile, it has been reported that the grazing can exert a negative effect on belowground biomass (Beaulieu, Gauthier, & Rochefort, 1996), yet there are also many studies indicating that the belowground biomass is equal in grazed areas compared with ungrazed areas (Derner, Boutton, & Briske, 2006; Garcia‐Pausas, Casals, Romanyà, Vallecillo, & Sebastià, 2011). To addresses these uncertainties and determine a suitable grazing intensity in alpine grassland, it is necessary to assess the effects of different grazing intensities on grassland ecosystems. On the other hand, grazing also has a profound effect on the vertical distribution of root biomass (Derner et al., 2006; Gill & Burke, 2002), but to date, very little work has explored the response of root biomass distribution to varying grazing intensity.

The Qinghai–Tibetan Plateau (QTP) is the main region of alpine meadow and alpine steppe, with an area of approximately 2.5 million km^2^ of alpine grassland representing one of the world's largest areas of alpine grassland (Dai, Guo, Du, Ke, et al., 2019; Dai, Guo, Zhang, et al., 2019; Dai, Ke, et al., 2019; Lu et al., 2017; Wesche et al., 2016). Most importantly, the QTP is the one of the most important natural pastures in China and has provided the main pastures for Tibetan communities for many years (Klein, Harte, & Zhao, 2007). In the past decades, the human population in the Tibet Autonomous Region has increased from 1.1 to 3.2 million, and livestock numbers have increasing by 0.3 million/year during 1951–2003 (Ma, Zhou, & Du, 2010). Meanwhile, the policy of excluding grazing in some specific regions has led to further increases in the concentrations of grazing outside such areas, resulting in overgrazing (Ma et al., 2010) and severe degradation or even thorough collapse of the alpine ecosystem (Harris, 2010). Currently, about 0.5 million km^2^ area of alpine grasslands on the QTP are severely degraded (Dong, Jiang, Zheng, & Zhang, 2012). The productivity of alpine grasslands has reduced by about 30% on the QTP during the last 20 years (Dong et al., 2012); this has not merely decreased the forage productivity, but also caused severe ecological problems such as declining water retention and increased soil desertification (Harris, 2010). Therefore, assessing the effects of different grazing intensities on plant communities (both species composition and plant biomass) is urgently needed in order to implement a suitable grazing intensity. Thus, the main objectives of this study were to (a) explore the effects of different grazing intensities on community biomass both aboveground and belowground, (b) observe the vertical distribution of root biomass under different grazing intensities, and (c) examine the relationship between productivity and species richness.

## 2 MATERIALS AND METHODS

### 2.1 Study area

This study was conducted in Huangcheng township, Haibei Tibetan autonomous prefecture of Qinghai province. The site has an average elevation of 3,230 m and is located on the northeastern QTP. The region has a characteristic plateau continental monsoon climate, with a mean annual air temperature of −1.7°C, and maximum and minimum air temperatures in July (9.8°C) and January (−14.80°C), respectively. The average annual precipitation is approximately 618 mm, with almost 80% falling in the growing season (i.e., from early May to late September; Dai, Guo, Du, Ke, et al., 2019; Dai, Guo, Du, Zhang, et al., 2019; Dai, Guo, Zhang, et al., 2019; Dai, Ke, et al., 2019). The soil is classified as Mat cryo‐sod soil on the basis of the Chinese National Soil Survey and Classification System (Institute of Soil Science, CAS, 2001).

**Figure 1:**
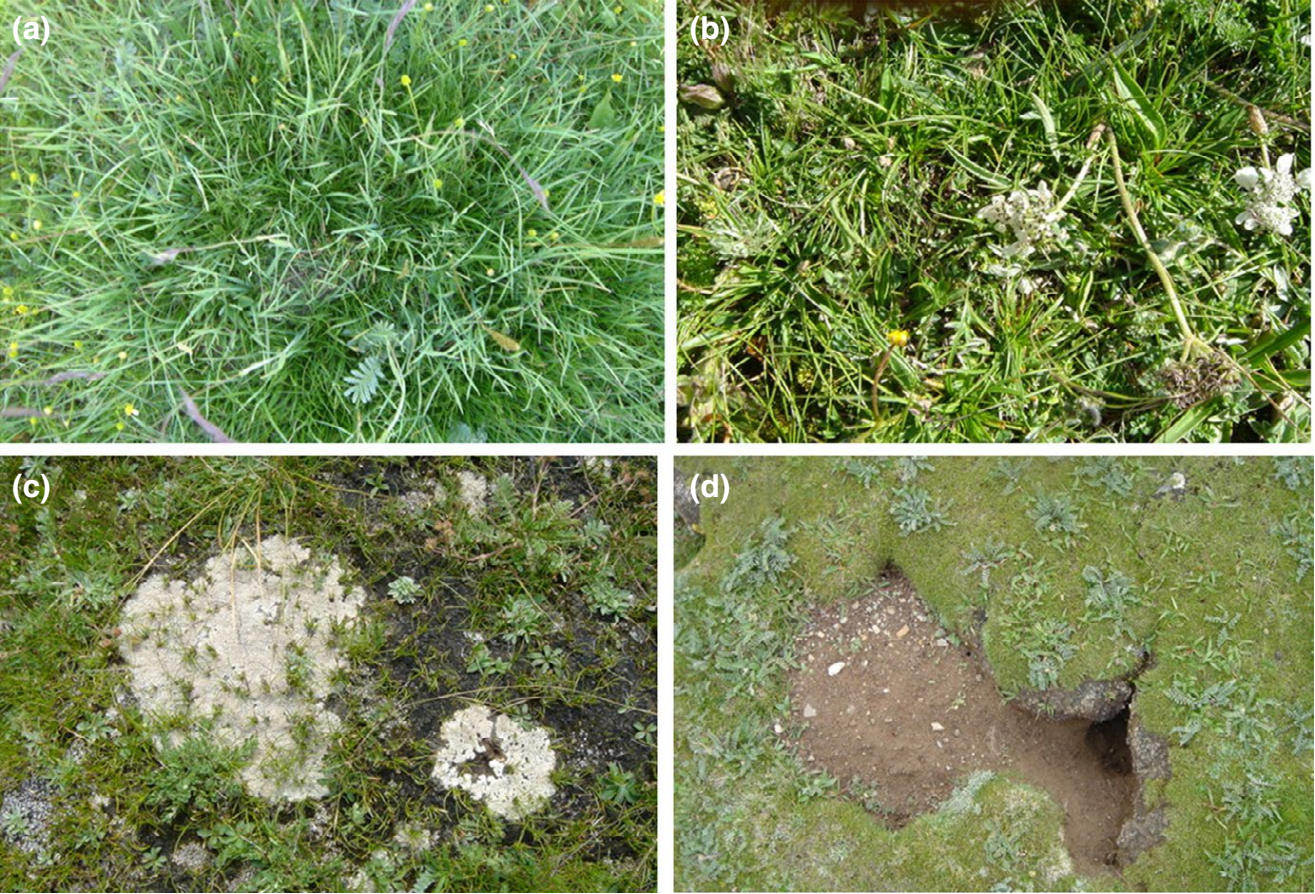
The degradation succession of *Kobresia* grasslands under four grazing intensity, (a) the Gramineae grass–*Kobresia humilis* community, (b) the *Kobresia humilis* community, (c) the thickening in mattic epipedon of the *Kobresia pygmaea* community, and (d) the cracks in mattic epipedon of the *Kobresia pygmaea* community

Before 1995, the vegetation distribution in this region is relatively uniform and not disturbed by grazing (the dominant species there being Grass and *Kobresia humilis*), and then, the pasture was assigned into four individual households after 1995. Thus, the composition of the plant community changed greatly as a result of different grazing strategies in the past 20 years, which formed four distinct degradation stages (Figure 1). According to the grazing intensity, four grazing patterns were formed: ungrazed (CK), light grazing (LG), moderate grazing (MG), and heavy grazing (HG), and correspondingly four typical communities were formed: (a) the Gramineae grass–*K. humilis* community, (b) the *K. humilis* community, (c) the thickening in mattic epipedon of the *K. pygmaea* community, and (d) the cracks in mattic epipedon of the *K. pygmaea* community (Figure 1), with one site in each pasture types having one intensity level of grazing. To exclude the discrepancy of climate and soil background values induced by spatial differences, the maximum spatial distance between the sample plots is <3 km. The grazing time is from December of the previous year to the end of April of the next year, which prevent the growing season of plant, and the grazing livestock are mainly Tibetan sheep. More details grazing information are provided in Table 1.

**Table 1:** Detailed information on the four grazing management schemes for alpine *Kobresia* grasslands

Grazing intensity	Stocking capacity	Location	Elevation (m)	Dominant plants
Ungrazed (CK)	—	37°39.02′N, 101°10.64′E	3,230	*Elymus nutans*
Light grazing (LG)	3.65 sheep**/**ha	37°40.16′N, 101°10.02′E	3,241	*K. humilis*
Moderate grazing (MG)	7.50 sheep/ha	37°40.05′N, 101°10.02′E	3,230	*K. pygmaea*
Heavy grazing (HG)	11.25 sheep/ha	37°42.09′N, 101°15.93′E	3,230	*K. pygmaea*

### 2.2 Data collection

We harvested the aboveground biomass (AGB) and belowground biomass (BGB) at the end of August (i.e., the time of peak biomass) in 2017 and 2018 across four grazing pattern sample plots (i.e., CK, LG, MG and HG). Three quadrats of 0.25 m^2^ (0.5 × 0.5 m) at 20 m intervals along the 100 × 10 m site across the four degradation plots, the AGB was obtained by a standard harvesting method, and four plant functional groups (sedge, grass, legumes, and forbs) were identified in each quadrat. The litter was also collected in each quadrat. The BGB was sampled by extracting 7‐cm diameter soil cores, with six replicates, from each quadrat at depths of 0–10, 10–20, 20–30, 30–40 and 40–50 cm. This was based on almost all root biomass being distributed in the top 50 cm (Cao, Du, Wang, Wang, & Liang, 2007). Samples were then cleaned to remove all soil particles. Finally, the AGB and BGB samples were oven‐dried at 65°C to a constant weight. In this study, the aboveground net primary productivity was considered as the annual peak biomass. The daily soil volumetric moisture and soil temperatures at 5 and 10 cm were measured by coaxial impedance dielectric reflectometry (Hydra probe II; Stevens) during the growing season of 2017 and 2018 (i.e., from early May to late September). The infiltration rate of soil water was measured by soil ring sampler with three replicates in each grazing pattern sample plots, the depth of mattic epipedon was measured by a ruler with three replicates in each grazing pattern sample plots.

### 2.3 Data analysis

Given the non‐normal data distributions of both AGB and BGB, the Wilcoxon rank test was used to compare the differences in biomass among the four grazing intensities, and a general linear model (GLM) was applied to examine the simple linear relationship between AGB and species richness across both the community and plant functional groups.

The vertical distribution of roots was characterized following Gale and Grigal (1987) by the asymptotic function, as follows:

$$Y=1-\beta^{d},$$

where β is the only estimated parameter in the model, and *Y* is the cumulative percentage of root biomass from the surface soil layer to depth *d* (cm). Values of *β* range from 0 to 1; high values of *β* indicate a greater proportion of deeper roots while a lower value of *β* indicates a greater proportion of roots near the upper soil surface. All statistical analyses were conducted in software package R (R Development Core Team, 2006).

## 3 RESULTS

### 3.1 Effect of grazing intensity on soil condition

**Figure 2:**
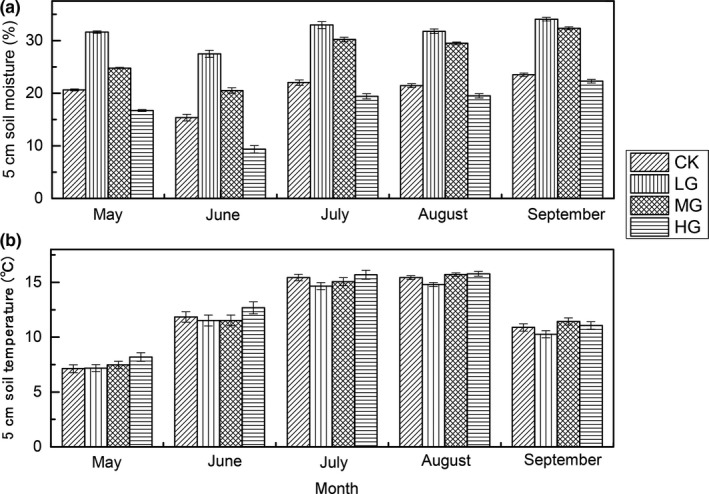
Seasonal variation of soil temperature and soil volumetric moisture during growing season across four grazing intensity

**Figure 3:**
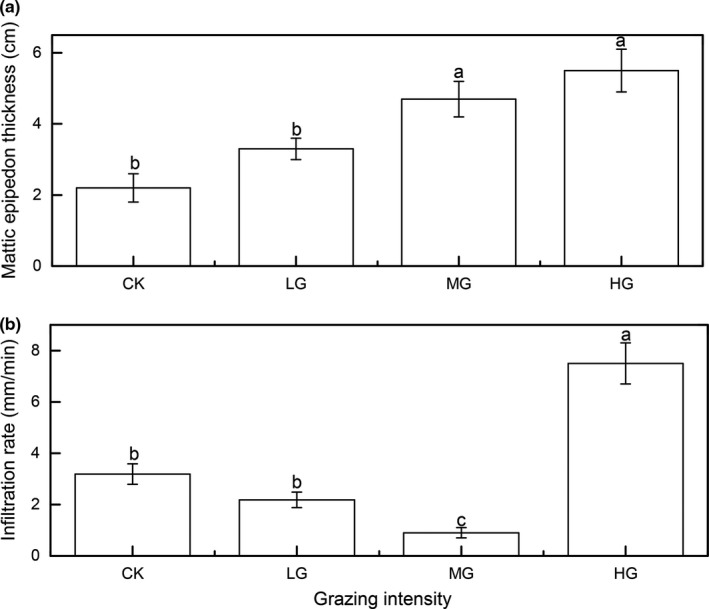
Comparison of mattic epipedon thickness and infiltration rate across four grazing intensity. Notes: different letters indicate significant differences between two grazing types (*p* < .05), the same below

The 5 cm soil volumetric moisture contents in LG were higher than CK from May to September, but then decreased with increasing grazing intensity during the growing season (Figure 2a), whereas the 5 cm soil temperatures increased with increasing grazing intensity during the growing season (Figure 2b). The thickness of mattic epipedon increased gradually with increasing grazing intensity (Figure 3a), and the thickness of mattic epipedon in MG was significant higher than CK and LG. However, the infiltration rate display decrease first and then rapidly increased trend (Figure 3b).

### 3.2 Effect of grazing intensity on aboveground biomass and belowground biomass

**Figure 4:**
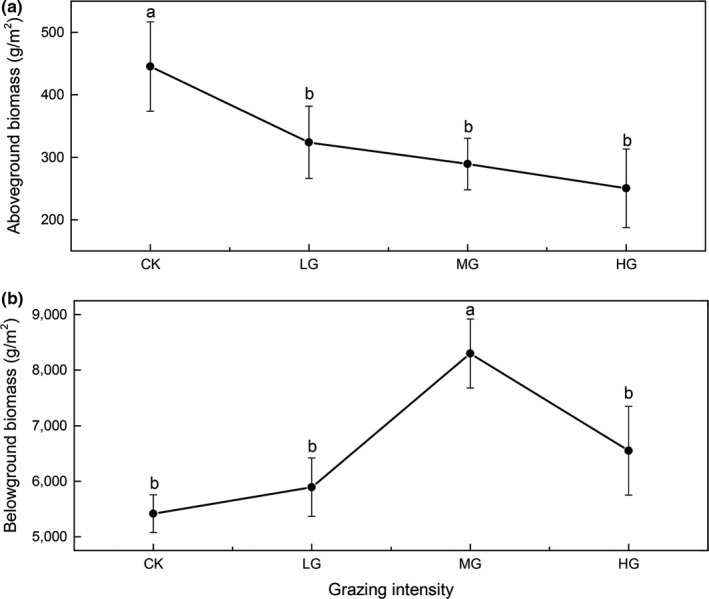
Comparison of aboveground and belowground biomass among four grazing intensity

**Figure 5:**
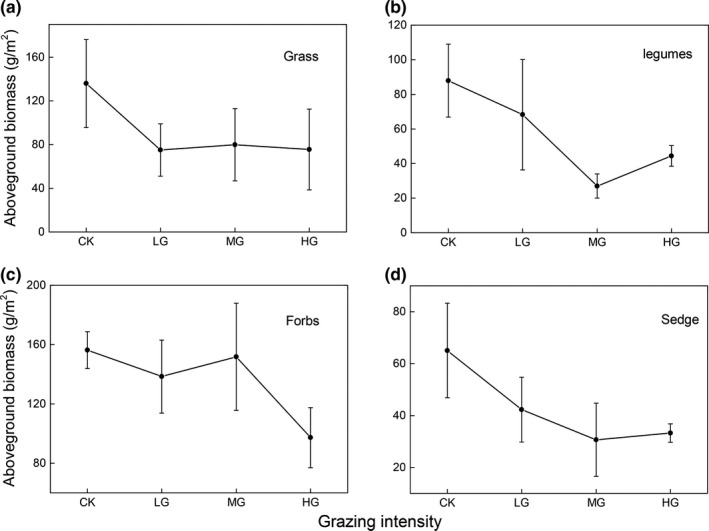
Effect of grazing intensity on the aboveground biomass of plant functional groups

The total AGB decreased with increasing grazing intensity. Moreover, the AGB in CK (445.15 ± 71.56 g/m^2^) was significantly higher than the AGB in LG, MG, and HG (323.94 ± 57.92, 289.24 ± 41.24, and 250.38 ± 62.81 g/m^2^, respectively; *p* < .05; Figure 4a). The functional group response to different to grazing intensities (Figure 5) showed that the AGB of grass decreased rapidly from CK to LG, then remained stable from LG to HG. Meanwhile, the AGB of legumes and forbs exhibited a decreasing trend from CK to MG, then slightly increased from MG to HG. There was no systematic change in forbs AGB from CK to MG, but it then decreased rapidly. In contrast, the BGB increased from CK to MG and then decreased, with the peak in MG (Figure 4b). The BGB in MG (8,297.72 ± 621.29 g/m^2^) was significantly higher than BGB in CK (5,414.65 ± 338.88 g/m^2^; Figure 4b).

### 3.3 Effect of grazing intensity on the vertical distribution of root biomass

**Figure 6:**
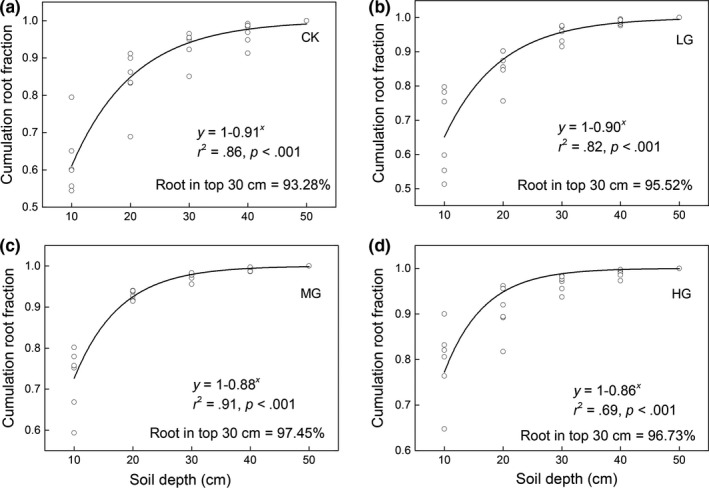
Vertical distributions of roots across four grazing intensity. The vertical distribution of roots across four grassland types was fitted by the function proposed by Gale and Grigal (1987)

**Figure 7:**
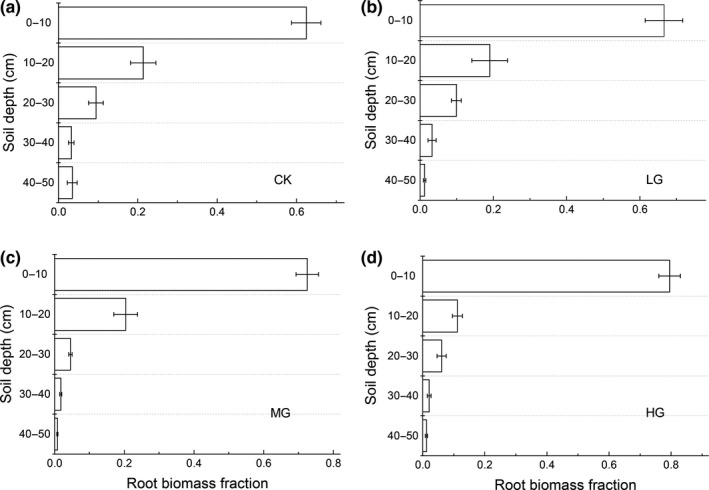
Root biomass fraction across different soil layers among four grazing intensity

Based on the asymptotic modeling of the vertical root distribution (Figure 6), more biomass was observed in the top soil layer of MG and HG, with 97.45% and 96.73% of roots in the top 30 cm of soil, respectively, compared to 93.28% and 95.52% for CK and LG, respectively. Furthermore, the *β* values for MG and HG (*β* = .88, *r* ^2^ = .91, *p* < .001 for MG; *β* = .86, *r* ^2^ = .69, *p* < .001 for HG) were lower than those of CK and LG (*β* = .91, *R* ^2^ = .86, *p* < .001 for CK; *β* = .90, *R* ^2^ = .82, *p* < .001 for LG), providing further evidence that MG and HG had shallower soil distributions than those of CK and LG. In addition to the root fraction, the root biomass decreased with soil depth across the four grazing intensities: the 0–10 cm root biomass fractions of CK, LG, MG, and HG were 0.62, 0.67, 0.73, and 0.79, respectively (Figure 7).

### 3.4 Productivity–species richness relationship and the impact of grazing intensity on species richness and litter

**Figure 8:**
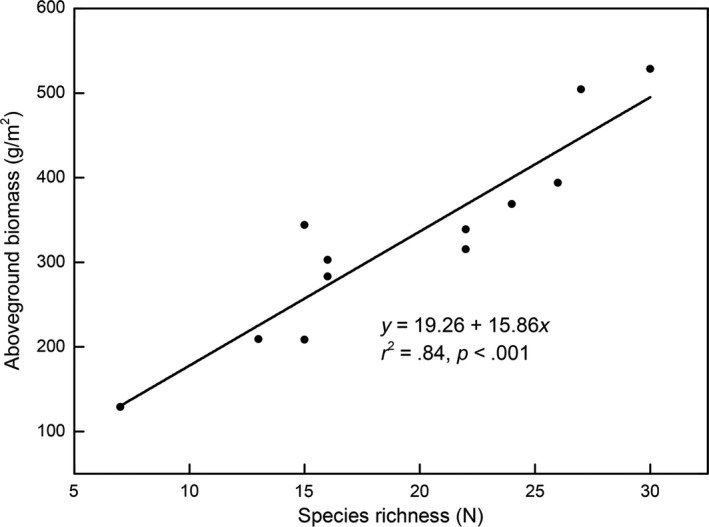
Relationship between species richness and aboveground biomass at community level

**Figure 9:**
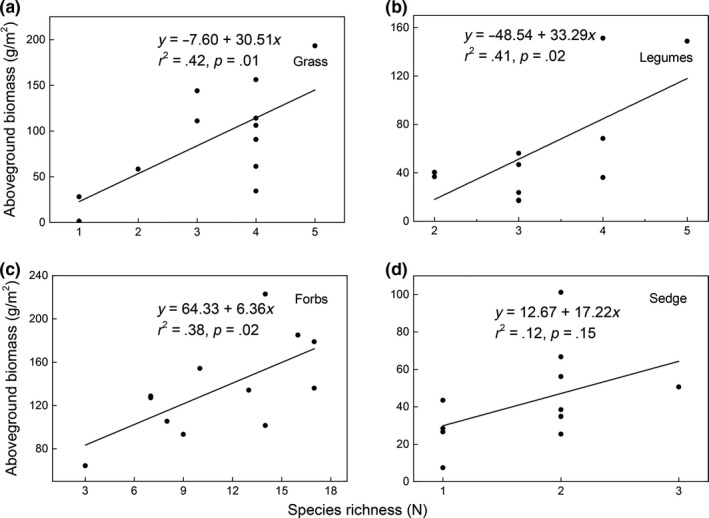
Relationship between species richness and aboveground biomass at plant functional groups level

**Figure 10:**
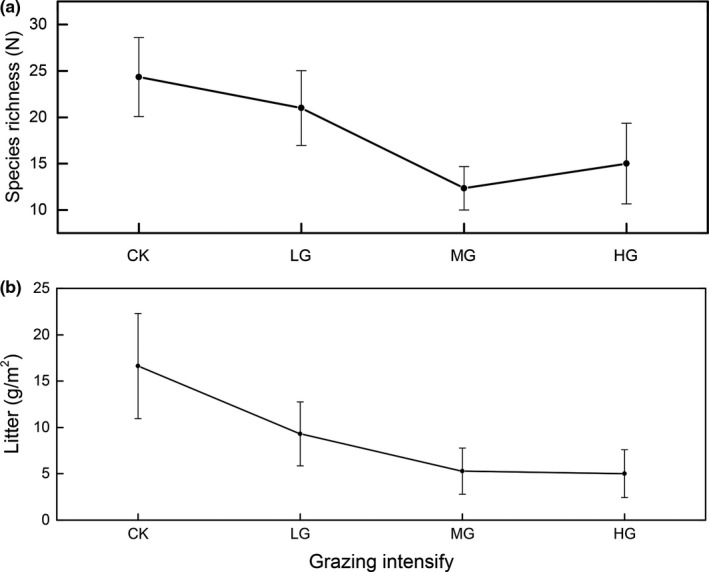
Effect of grazing intensity on species richness and litter

There was significant positive relationship between productivity and species richness (*r* ^2^ = .84, *p* < .001), both at community and at functional group levels except for legumes (*p* < .05; Figures 8 and 9). Moreover, the species richness decreased from CK to MG and then increased, with the lowest species richness occurring in MG (Figure 10a). The litter amount decreased with increasing intensities of livestock grazing (Figure 10b).

## 4 DISCUSSION

### 4.1 Effect of grazing intensity on aboveground biomass

The productivity and plant biomass are important indicators of ecosystem structure and function in alpine grasslands. We found that the AGB decreased with increasing sheep grazing intensities (Figure 2a). The negative effects of sheep grazing on AGB might be attributed to how the plant functional groups respond to grazing (Fu, Shen, Zhang, Zhou, & Zhang, 2012; Wu, Shang, Zhu, Ding, & Wang, 2016). Obviously, the sheep grazing reduces AGB through the consumption of vegetation for feed, in particular for grass and sedge species (Shi et al., 2013). Furthermore, the AGB of grasses decreased the most from CK to LG, followed by sedge and legumes (Figure 3). This pattern could be explained by the bilayer structure in the CK and LG communities, in which the upper layer was mainly dominated by grass plants such as *Stipa *spp. and *Festuca *spp., while the lower layer was mainly dominated by sedge and legumes. Since the sheep preferentially feed on upper plants (i.e., grasses) due to the livestock preferentially defoliation the upper plant, sheep grazing caused the AGB of grasses to be reduced faster from CK to LG than that of the other two functional groups. The AGB of grass and forbs showed slight increases from LG to MG, while the AGB of legumes and sedge decreased continuously to their minimum values (Figure 3).This may be attributed to the single layer structure in the MG community, which is mainly dominated by sedge and legumes with grass species occurring less commonly; thus, the sheep may select more sedge and legumes for feed, leading to a continuous declination in sedge and legumes but little change for grass (Lin et al., 2015). Finally, after the sedge and legumes were almost completely removed, the sheep had to feed on the forbs, resulting in a sharp reduction from MG to HG for forbs, while the AGB of the sedges and legumes began to increase gradually.

Owing to increasing water stress in the plants, the reduce of AGB may also be associated with the deteriorating soil conditions under increased grazing intensities, such as soil moisture loss (Tang, Zhao, & Zhou, 2006; Yates, Norton, & Hobbs, 2000). Another possible mechanism related to the decrease in AGB under sheep overgrazing might be related to the amount of litter, because litter has some water holding capacity that can reduce soil water evaporation (especially during summer, under stronger radiation; Zhang, Qi, et al., 2014). Our study indicated that the litter amount decreased with higher intensities of sheep grazing (Figure 6b); consequently, the surface soil temperature increased (Figure 1). The higher temperature in the surface soil could reduce AGB by reducing the surplus soil moisture available for plants via evaporation (Zhang et al., 2018). Therefore, we might conclude that the AGB decrease was induced by sheep overgrazing intensity, combined the effects of soil moisture and the response of functional groups to sheep overgrazing intensities.

### 4.2 Effect of grazing intensity on belowground biomass and its vertical distribution

In contrast to AGB, the BGB increased with sheep grazing intensity from CK to MG with the maximum value in MG (Figure 2b). First, these discrepancies may reflect differences in soil condition and species composition. For instance, the development of mattic epipedon (felty fine dead and living roots) due to the unique biological characteristics of *Kobresia pygmaca* (i.e., its very high root: shoot ratio) could lead to the increasing BGB from CK to MG. Second, the poor nutrient conditions, due to low amount of mineralization, may stimulate the allocation of root biomass as a strategy for absorbing more soil nutrients (López‐Mársico, Altesor, Oyarzabal, Baldassini, & Paruelo, 2015; Lu et al., 2017). Meanwhile, a previous study suggested that cattle grazing trends to enhance BGB by decreasing allocation to aboveground parts and increasing the BGB allocation to resist grazing pressure and aid germination (López‐Mársico et al., 2015). Last but not least, in order to restore growth, the plant might allocate more photosynthetic products to its belowground parts to obtain more nutrients and water (Frank, Kuns, & Guido, 2002). This would explain the increase in BGB from CK to MG, however, a declining trend in BGB was observed from MG to HG, perhaps owing to the crack in mattic epipedon by livestock trampling and soil freeze–thaw (Figure 2). There is a growing evidence indicated that the mattic epipedon has a certain capacity of water retention and prevent most rainfall return to atmosphere through evapotranspire (Jing et al., 2015; Zhang, Wang, et al., 2014), thus more soil water could be obtained for the root growth. However, once the crack format in mattic epipedon due to livestock trampling, the soil water infiltration could be exacerbated (Figure 3a), leading to most soil moisture and nutrients was not captured by root, ultimately resulting in the alpine grassland degraded to black soil beach. Combining these factors, we might conclude that the mattic epipedon play a vital role in regulating the alpine ecosystem stability via altering the soil water and nutrients condition, consequently altering the plant community characteristics. Therefore, we suggest that the moderate levels of disturbance may optimize grassland management for alpine meadow in the case of root production, especially considering that the important role of mattic epipedon in maintain the stability of alpine ecosystem.

Furthermore, the vertical distribution of root biomass was also greatly shaped by the intensity of sheep grazing. We found that the root distribution became shallower as grazing intensity increased, consistent with previous studies showing that mesic communities under livestock grazing condition tended to allocate more biomass in the uppermost soil layers (Rodríguez, Brown, & Gómez‐Sal, 1995). It is possible that the shallower distribution of root biomass under sheep overgrazing might be attributed to the concentration of nutrients in the soil surface resulting from sheep dung and urine (Stumpp, Wesche, Retzer, & Miehe, 2005). Given that the alpine ecosystem is limited by low temperature, leading to reduced nutrient mineralization and consequently fewer soil nutrients near the soil surface (especially nitrogen that limits alpine plant growth), plants were more sensitive to these additional nutrients. It is well known the amounts of sheep dung and urine increase in the surface soil layer as sheep grazing intensity increases (Stumpp et al., 2005), resulting in greater biomass allocation near the soil surface in order to obtain more soil nutrients. An alternative explanation for the shift in root biomass to shallower soil layers under overgrazing conditions might relate to changes in species composition: the proportion of root biomass in deeper layers decreases when their species richness is reduced and is positively related to ANPP (Mueller, Tilman, Fornara, & Hobbie, 2013). Moreover, the covariance between root depth distribution and plant biomass was not only dependent on plant species richness, but also on the presence of plant functional groups. Several studies have shown that deeper roots are often associated with legumes and sedge biomass (Mueller et al., 2013); here, our results (Figure 5) show that the legumes and sedge AGB both decrease with increasing sheep grazing intensities (except for HG). Therefore, the reduced legumes and sedge biomass might lead to the shift of root biomass to shallower soil layers under grazing conditions, when considering the good correlation between species richness and AGB (Figure 7). Overall, the effects of sheep grazing on the vertical distribution of root biomass may be largely mediated by the combined effects soil nutrients and species composition of the aboveground community.

### 4.3 The relationship between species richness and productivity

The relationship between species richness and productivity has been a central but controversial issue in plant ecology for a decade. A growing number studies have documented the productivity–richness relationship (Bai, Han, Wu, Chen, & Li, 2004; Gao, Men, & Ge, 2014), mostly finding that a unimodal relationship at scales from local to landscape, although several studies have found no correlation between species richness and productivity (Fraser et al., 2015; Gao et al., 2014). Here, we found a positive relationship between species richness and AGB across both community and all the plant functional groups except for sedges (Figures 6 and 7).This is promoted to some degree by the greater gains in soil resources in more diverse plant communities (Fornara & Tilman, 2008). Firstly, more diverse plant communities have a greater likelihood of including high‐yielding nitrogen species (such as the N‐fixing legumes) which capture more nutrients needed for the growth of the aboveground part of plants; this is important since most terrestrial systems are limited by N availability, in particular alpine ecosystems (Vitousek & Howarth, 1991). Secondly, facilitation and complementarity are often considered as important factors in the positive productivity–species richness relationship (Hooper et al., 2005). For instance, the complementarity could decrease the interspecific competition via niche partitioning, to provide more available resources for the plant community (van der Maarel & Titlyanova, 1989); indeed, many previous studies have found that higher species richness can enhance the rates of ecosystem N cycling by positively affecting the microbial community (Zak, Holmes, White, Peacock, & Tilman, 2003), and some plant species exhibit a higher N‐use efficiency when species diversity is higher (Fargione et al., 2007). Furthermore, facilitative interactions among species could increase growth rates or expand ecosystem resource pools as the species richness increases, thereby helping to alleviate harsh environmental conditions or facilitating other species to capture critical resources (Mulder, Uliassi, & Doak, 2001).

### 4.4 Implications for local people and government policy

In the recent decade, the grassland ecosystem on the Qinghai–Tibetan Plateau has been experiencing significant changes of degradation and restoration under both climate change and human activities, especially enforcement of unreasonable policies from central and local government. Sheep grazing practiced by Tibetan herders for many centuries is considered a major human activity in the alpine grasslands, which in the long run lead to overgrazing, inducing serious degradation in the alpine grasslands. Thus, there is a sense of urgency to determine a suitable stocking rate in the alpine grassland for restoring degraded grassland and improving livestock production both at the local and at the landscape scale (Barcella, Filipponi, & Assini, 2016; Metera, Sakowski, Słoniewski, & Romanowicz, 2010). In this study, we found that the maximum value of BGB occurred in moderate levels of grazing disturbance (i.e., 7.5 sheep/ha), suggesting that such stocking rates may be an optimal grassland management strategy for the alpine meadow in terms of root production, especially considering the BGB almost accounts for more than 80% biomass of plants (Dai, Guo, Du, Ke, et al., 2019; Dai, Guo, Zhang, et al., 2019; Dai, Ke, et al., 2019), which could provide new insight for local people and policy‐makers in government. However, it should be noted that the species richness shows the lowest value in MG in spite of the BGB peaked in MG. This result indicated that there may be a trade‐off between species richness and root production in grazing management, and thus appropriate government policy should be implemented. Because the changes in grazing practices are affected by socioeconomic developments, part of the income loss induced by the implementation of a low‐intensity grazing management should be compensated by agri‐environmental schemes. Furthermore, the selection of livestock type also has a profound impact on the plant community. A previous study found that the sheep is a more selective grazer compared to the cattle (Tóth et al., 2018). For instance, compared with cattle grazing, sheep grazing maintains a lower functional diversity and taxonomic, lower number of forbs (Tóth et al., 2018). Therefore, it is inferred that the livestock type may play a more important role than grazing intensity; this result was also reported by a previous study (Tóth et al., 2018). In this study, we just discuss the impact of sheep grazing on plant community, and we suggest that more livestock types should be combined to have a better understanding of the optimal grazing pressure for maintaining biodiversity in the alpine grassland.

## 5 CONCLUSIONS

Grazing is one of the most important disturbance factors leading to the grassland degradation, and the subsequent decline in productivity and ecological function of plant communities. Our results show that the AGB was reduced by increasing grazing intensity, but that individual plant functional groups showed varied responses to the different intensities of livestock grazing. Meanwhile, species richness decreased with increasing grazing intensity from CK to MG, with the minimum in MG, before then increasing in HG. In contrast to AGB, the BGB exhibited a unimodal relationship with a maximum in HG, implying that moderate levels of disturbance may be an optimal grassland management strategy for alpine meadow in terms of root production. Plants tended to allocate more biomass to shallow roots under increasing grazing intensities; this shift in root biomass toward the top soil layers under grazing may affect the stability of soil organic carbon in the subsoil. Our results reflect the asymmetrical responses of aboveground and belowground biomass to different grazing intensities, and could aid in grassland conservation and its appropriate management.

## CONFLICT OF INTEREST

The authors declare no conflict of interest.

## AUTHOR CONTRIBUTION

L Dai performed the research, analyzed data, and wrote the paper; F Zhang, X Guo, X Ke, Y Li, Y Du, C Peng, L Lin, Q Li and K Shu analyzed data; G Cao conceived the study.

## Data Availability

The biomass and other data in this paper are available in Dryad: Dryad https://doi:10.5061/dryad.1pr59gf.
